# *QuickStats:* Birth Rates* for Teens Aged 15–19 Years, by Age Group — National Vital Statistics System, United States, 1991–2018

**DOI:** 10.15585/mmwr.mm6840a7

**Published:** 2019-10-11

**Figure:**
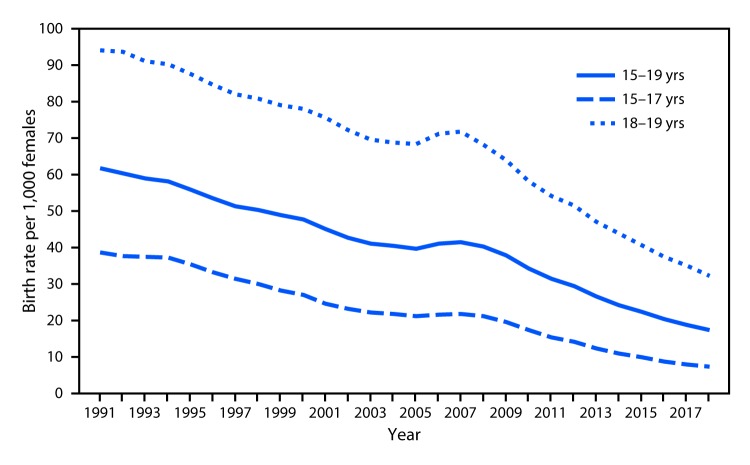
The birth rate for teens aged 15–19 years declined from a peak of 61.8 per 1,000 females in 1991 to a record low of 17.4 in 2018. The rate has declined more rapidly since 2007. From 2007 to 2018, the rate declined from 21.7 to 7.2 for teens aged 15–17 years and from 71.7 to 32.3 for teens aged 18–19 years.

Source: NCHS, National Vital Statistics System. Birth Data, 1991–2018. https://www.cdc.gov/nchs/nvss/births.htm.

